# The short-term effect of a myofascial protocol versus light touch applied to the cervical spine towards the prevention of balance disorders in the elderly: protocol of a randomised controlled trial

**DOI:** 10.1186/s12998-022-00446-0

**Published:** 2022-08-31

**Authors:** Laurianne Pinloche, Solène Souvignet, Michèle Germain, Karine Monteil, Christophe Hautier

**Affiliations:** 1 ISOstéo Lyon, Unité Recherche, Lyon, France; 2 Hospices Civils de Lyon, Hôpital de La Croix Rousse, Lyon, France; 3 LIBM, Université Claude Bernard Lyon 1, Lyon, France

**Keywords:** Older adults, Balance, Gait, Myofascial release, Neck, Strength, Mobility

## Abstract

**Background:**

Falling is a major trauma that can occur with aging, leading to very significant psychological and physical health effects with financial and societal consequences. It is therefore essential to explore therapeutic treatments that can reduce this risk. Some recognized effective treatments exist, concerning in particular the re-education of the muscles of the lower limbs. However, to our knowledge, none of them focus on the cervical spine although the latter is located at an essential physiological crossroads. Manual therapy, which has already demonstrated its impact on pain and balance parameters in the elderly, could be a painless and non-invasive tool of choice in addressing this problem.

**Methods:**

Interventional study (not related to a health product), monocentric, prospective, controlled, randomized double-blind (patient and evaluator performing the measurements). The experiment will take place over three measurement periods on D0, D7 and D21. On D0 subjects will be randomized in 2 groups: experimental and placebo group. Both groups will be assessed on: Short Physical Performance Battery test score, walking speed, lower limb strength, balance, heart rate variability and cervical spine strength and mobility. Then the experimental group will receive a myofascial release protocol applied to the cervical spine and the placebo group will receive a placebo light touch protocol. The intervention will be followed by the same measurements as before. This schedule will be reproduced on D7. On D21, only one assessment will be done.

**Discussion:**

This study started in 2020 but could not go beyond the inclusion phase due to the COVID pandemic. It is envisaged that recruitment could resume during 2022.

*Trial registration*: Registered by the Comité de Protection des Personnes—Sud Méditerranée; under the title “Prévention des troubles de l’équilibre chez le senior: influence de la thérapie manuelle appliquée au rachis sur les paramètres statiques et dynamiques», n° 19.12.27.47.259 in date of February 4, 2020.

Registered by ClinicalTrials.gov ID: NCT05475652; under the title « The Influence of Manual Therapy Applied to the Cervical Spine in the Prevention of Balance Disorders in the Elderly (ManEq)”.

## Background

Falls represent a major health problem in elderly subjects because of their high prevalence and the seriousness of their physical, functional, psychological and financial consequences. Indeed, about 30% of independent people living in the community over 65 years old and 50% of those over 80 years old suffer at least one fall per year; a third of the fallers experience repeated falls [1]. The human and economic cost of falls increases along with the aging of the population, particularly in Western societies. In 2015, EuroSafe (European Association for Injury Prevention and Safety Promotion) [2] classified falls as the 3rd most common cause of disability among the elderly and one of the main causes of admission to specialized institutions.

Prevention of falls is widely recognized as one of the priorities for healthy and active aging of the population. Most recommendations for fall prevention, such as STEADI [3] or The ProFouND [4], call for regular assessments of elderly patients at risk and the provision of appropriate interventions. Van Voast Moncada et al. [5] summarized the different interventions evaluated in the literature, such as therapeutic education, dietary supplementation, functional rehabilitation to improve walking, strength or balance, treatment of hypotension, review of medication, podiatry or even management of vision problems or home design. Taken separately, each intervention has only a moderate impact on the risk of falling, and elderly people at risk of falling should be offered a broader protocol combining different methods [6]. Prevention of physical deconditioning is a prerequisite for "successful aging" [7]. Various elements have been identified as physical components [8] that contribute to the maintenance of autonomy and can therefore be used as a control during management. In elderly people who fall, there is a maladaptation of the balance strategy: weakened postural and general control, impaired visual feedback, as well as impaired proprioceptive and vestibular systems and neuromuscular coordination [9, 10]. The loss of lower limb strength directly influences Walking Speed (WS) [11], which is a recognized marker for predicting the risk of falling and is correlated to static and dynamic balance [12]. Age leads to the appearance of a cautious attitude with a reduction in WS [13] and a change in the distribution of support on the ground with an increase in pressure peaks at the level of the hindfoot and a decrease in support at the level of the hallux [14–16].

Spinal curvatures change with age, tending to a general increase in curvatures, an increase in asymmetries and an protraction of the head [17, 18] which may influence balance in the senior citizen. The neurological system also undergoes major modifications with a reorganization of the motor units [19], a decrease in the number of mechanoreceptors, and an alteration of the function and vestibulospinal reflexes [20]. Finally, the aging of the autonomic nervous system and more particularly of its parasympathetic component [21] may indirectly influence balance through its link with the cardiovascular system [22]. This is why the analysis of Heart Rate Variability (HRV) is a good indicator in the evaluation of the risk of falling due to orthostatic decrease in the elderly [23].

From a clinical point of view, the cervical spine could be of particular importance due to its position and constitution: at crossroads of articular, visual and vestibular proprioceptive afferents, rich in neurological and motor elements [24–26]. Its role is mechanical (shock absorption of movements [27] and stabilization of vision [28]); but also neurological (regulation of the autonomic nervous system). Quek et al. [29], have shown that a decrease in cervical mobility and the presence of rotational asymmetry caused an alteration in anteroposterior oscillations during static equilibrium. They hypothesize that in subjects unable to effectively recruit the muscular and proprioceptive system of the neck, a compensatory system based on vision and vestibular system comes into play. This suggest that preserving cervical movement capacity across lifespan could limit compensations and risk of falling. The role of the tone of the cervical musculature seems to be essential since it has been shown [30] in young subjects that muscular fatigue in that area reduces the speed of displacement of the Center of Pressure (CoP), and in elderly subjects there is a modification of the recruitment pattern of the neck muscles in favour of the superficial muscles (to the detriment of the deep muscles) which favours the modification of the cranio-cervical angle and the orientation of the lordosis, generating an protraction of the head [31].

If the management of the cervical spine seems to be of interest in balance strategy, its vascular and neurological environment makes investigation and local treatment delicate especially in elderly. Myofascial Release (MFR), which is a form of manual therapy, seems particularly appropriate as it is non-invasive and can be adjusted to the recommendations of use. MFR needs application of a low load, long duration stretch to the myofascial complex, intended to restore optimal length, decrease pain, module neural transmission and improve function. The effects of neuromuscular stimulation of the cervical region remain poorly explored. Studies have shown that the application of vibrations to the cervical spine improves sensorimotor function and decreases postural sway in subjects with neck pain, while it alters head positioning in healthy subjects [32, 33]. Applied on C4 level this technique could provide stimulation of the carotid sinus and its baroreceptors, the superior cervical ganglion, the nuchal ligament, the longus neck muscle and the vestibulospinal reflex. An other technique commonly applied in MFR, usually called “suboccipital decompression”, seems to have an interesting impact on different postural determinants, in particular the cranio-cervical angle, the position of the head or even the stiffness of the posterior chains of the lower limbs [34, 35]. This technique enables an action on the oculo-cephalogyre muscles (the rectus capitis posterior major and minor, obliquus capitis superior and inferior) and the vestibulospinal reflex as well as the upper cervical proprioceptors, the neurovegetative structures of the posterior torn hole (X and XI) and of the carotid corpuscle by anastomosis. Other manual protocols aimed at the myofascial system as a whole or more specifically in the neck region have been evaluated and seem to indicate a favourable effect on balance, especially on anteroposterior sway [36] which is a parameter characterizing the displacement of the CoP, cervical mobility [37, 38] or even the Time Up and Go Test (TUGT). Unfortunately, these studies are sometimes of questionable methodological quality whether this is because of a small number of subjects, the absence of a controlled or placebo group or even a timing of different treatments depending on the groups.

However, there is no consensus on the frequency or number of treatments necessary with MFR, regardless of the reason for which they are used, for example: chronic low back pain, fibromyalgia, functional capacities… [39, 40]. Whatever the modality of application (massage or osteopathic manual therapy) or location (lower limb or trunk) it seems that only one MFR session have a limited or even non-existent effect on balance [41, 42] while recent studies have shown that two sessions can [43, 44].

To our knowledge, no study has yet proposed to evaluate the effect of a cervical spine MFR protocol on balance in older adults. The main hypothesis of the present study is that a two sessions MFR protocol have a beneficial impact on the balance and motor performance of older adults, which will result in improved balance and WS in the experimental group compared to the placebo group. The study also aims to propose an assessment of the physical parameters of balance, looking for links to validate the contribution of the cervical spine in compensatory mechanisms.

### Primary and secondary objectives

The primary objective of this work will be to evaluate the influence of some MFR techniques address to cervical spine on WS and SPPB (Short Physical Performance Battery test) score. It can be assessed by the main hypothesis:

H0: SPPB score and WS of MFR protocol group ≤ SPPB score and WS of light touch group.

H1: SPPB score and WS of MFR protocol group > SPPB score and WS of light touch group.

Secondary objectives will be to evaluate the influence of the protocol on other measured balance parameters, including cervical strength and mobility as well as lower limb strength, ground support and HRV. The data can also be used to explore the relationships between the different parameters.

## Methods

### Design

A two-armed (sham group and intervention group) randomized controlled trial designed according to the SPIRIT guidelines [45].

### Setting

This study will take place in a single center, located at the Croix Rousse Hospital in Lyon, France.

### Participants

#### Inclusion criteria

Volunteers for the study will be included if they are 65 years of age or older, of either sex, autonomous, able to walk ten meters alone without walking aid and able to understand instructions necessary for the correct performance of the measurements.

#### Exclusion criteria

Potential participants will be excluded from this study if they are suffering from a locomotor handicap or severe chronic progressive pathologies preventing the protocol from being carried out correctly, if they have a history of surgery, fracture or dislocation of the cervical spine, if they are institutionalized, if they have cognitive impairments (Mini Mental Statement < 20) or that their life expectancy is less than 6 months. Protected individuals: adults under curatorship or legal protection, deprived of liberty by judicial or administrative decision cannot be included.

#### Recruitment

Participants will be recruited from the local population by announcement. The study will be advertised on posters displayed at town halls, senior gyms, senior associations, pharmacies. Those who apply will be referred to a therapist who will make sure all eligibility criteria are met and give more information.

### Study schedule

#### Screening

On the day of the experiment or before, the volunteer will be welcomed by the principal investigator. After ensuring that the information provided previously has been properly understood, the investigator will present the premises and introduce the experimenters and then seek their written consent to participate in the study. An inclusion visit will also be carried out by a doctor. If they accept, the volunteer will sign the informed consent form before the study is carried out.

The participant can ask to leave the study at any time. They will be given an information sheet including the contact details of whom to approach in the event of any additional request or to report any adverse events or new facts. Finally, an SF-36 questionnaire is given to complete at home and bring back on D7.

**Fig. 1:**
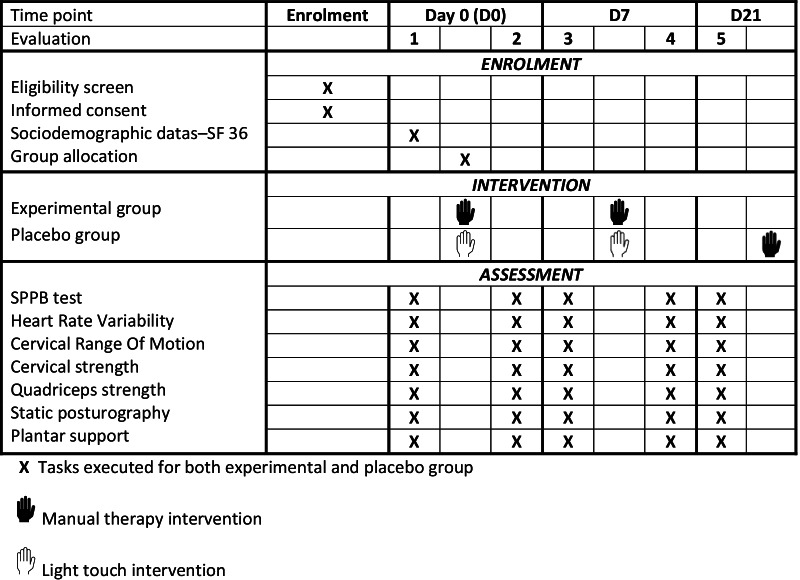
Study schedule summary

The study design, which covers the screening, evaluation, intervention, and follow-up procedures is summarized in Fig. 1.

#### Randomization and blinding

This study will use a process of allocation concealment which means that recruitment and randomization will be separated. The randomization will take place after the first session of measurements on day 0, just before the interventional part. Patients will be allocated to two groups, a sham group and an experimental group, in sequential order. Opaque envelope, prepared in advance, containing the group assignment, will be drawn by lot by the single therapist. This last will remain blind for the results obtained during evaluation and will be responsible for all the interventions in both groups. Similarly, the evaluator responsible for data collection will be blinded to the treatment allocation group (intervention or sham light touch). The placement of the hands and the timing of the techniques being strictly the same in both cases, the evaluator has no way of knowing which treatment has been applied. Subjects giving their informed consent are informed that a placebo treatment may be administered to them, and that they will not know which treatment has been applied until the end of the study. They are also told that the manipulations received are to be gentle, applied with light pressure and that they will therefore be unable to tell the difference. The subject will be questioned following the last series of measurements on their perception of the protocol received: placebo or experimental.

#### Intervention

The experiment will take place over three measurement times on Day 0 (D0), D7 and D21. On D0 subjects will be randomized in 2 groups: experimental and placebo group. Both groups will be assessed on: SPPB, WS, lower limb strength, balance, HRV and cervical spine strength and mobility. They will also be given an SF 36 questionnaire (to complete at home and bring back to D7). Then the experimental group will receive a MFR protocol applied to the cervical spine and the placebo group will receive a placebo light touch protocol. The intervention will be followed by the same measurements as before. This schedule will be reproduced on D7. On D21, only assessment will be done. Measurements will be made by the same experimenter for all sessions for the same patient. The time of presence required is estimated at 4 h maximum: approximately 1h30 on D0 and D7 and 45 min on D21.

The MFR protocol applied to the cervical spine in this study is carried out by one therapist—Osteopath D.O. (Diploma in Osteopathy)- with one year of experience. All the gestures performed and described below are manual osteopathic techniques with myofascial aim, mastered and commonly used in osteopathic practice management. Whatever the patient's allocation group, the techniques are gentle and progressive, the patient's head (lying on his back) remaining in line with the spine.

**Fig. 2:**
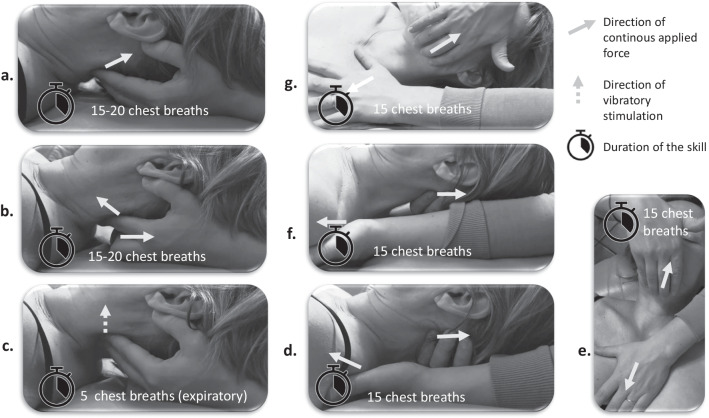
The manual therapy protocol of the study in 7 skills. **a** Suboccipital decompression; **b** Myofascial disengagement of Occiput, Atlas, Axis; **c** Vibratory stimulation on C4 level; **d** Myofascial release of pre-vertebral deep cervical aponeurosis; **e** Myofascial release of middle cervical aponeurosis; **f** Myofascial release of superficial cervical aponeurosis, posterior part; **g** Myofascial release of superficial cervical aponeurosis, anterior part

For the treated group, initially, the manual therapist, seated at the head of the patient, will perform a “suboccipital decompression” [46]—Fig. 2a. To do this, he will position the last 4 fingers of each hand towards the lower part of the occiput, the last phalanges in contact with the sub-occipital muscles. He will look for a so-called “bone contact” on the occiput. He will apply anterior and superior continuous pressure during 15 to 20 chest breaths.

As a 2nd step, the practitioner will carry out a “disengagement of Cervical 0 (C0)-C1-C2” [37, 47]—Fig. 2b. For this, the 4th and 5th fingers will be positioned on the scale of the occiput in its lowest part, index fingers on the spinous of C2 as shown in Fig. 15, thumbs along the mastoids. Anterior tension will be applied on C2, a cephalic strain on the occiput and a caudal pressure on C2. The action will be maintained for 15 to 20 chest breaths.

As a 3rd step, the manual therapist will perform a “vibratory stimulation at C4 level”[38, 48, 49]—Fig. 2c. To do this, the 2nd and 3rd fingers will be positioned opposite the posterior part of the C4 transverses and the 4th and 5th fingers at the level of the lower part of the occiput. Cephalic traction will be performed at the level of the occiput by bending the last 2 fingers and vibrations will be applied to the expiratory phases by the index and middle fingers on either side. This action will be performed over 5 breath cycles.

As a 4th step, the practitioner will perform a “myofascial release technique of the cervical aponeuroses” [50–55]. For the pre-vertebral deep cervical aponeurosis, one hand is placed at cephalic level at the base of the occiput exerting cephalic traction, the other hand is placed at the level of the posterior surface of Thoracic 4 (T4) and exerts anterior pressure and caudal traction—Fig. 2d. For the middle cervical aponeurosis, one hand is placed on the lower part of the mandible and exerts cephalic traction and the other at the level of the sternal manubrium and exerts caudal traction—Fig. 2e. Swallowing is required on exhalation. For the superficial cervical aponeurosis, the hands will be placed successively: at the level of the upper occipital curved line and on the right, then the left scapular spine; then at the level of the lower edge of the mandible and the upper edge of the homolateral clavicle, on one side then on the other—Fig. 2f, g. The 2 hands exert opposing pulls. Each stretch is done over 15 breathing cycles.

For the placebo group, the patient will receive a so-called "light touch" treatment which consists of applying light pressure with broad support from both hands on either side of the joint, in contact with the skin, without equal bone pressure or therapeutic intention, for a duration comparable to interventional group. Thus, the positions of the placebo treatment will be the same as previously described for the group treated with MFR so that the credibility of the placebo treatment is acceptable, with the difference of less manual pressure applied: only in contact with the skin.

## Outcomes

### Primary outcome

The primary outcome of this study will be WS and the SPPB test score.

### Secondary outcome

The secondary outcomes will be as follows: HRV, cervical range of motion, cervical muscle strength, quadriceps strength, static posturography and plantar support.

### Data collection

All data except HRV and posturographic data will be collected and noted by the evaluators on paper records specific to the evaluation. HRV, posturographic and plantar data will be collected and automatically stored in individual file folders (secure by password) in a computer belonging to University Claude Bernard Lyon 1 (UCBL1) used exclusively for the study and protected by password too. After the conclusion of the data collection, the data will be transcribed in an Excel® spreadsheet by an examiner using codes to preserve volunteer identification. All data will be saved on an encrypted UCBL1 server. Synchronization between the computer and the server will be ensured by the software Druva inSync ®.

### Method for data collection

The timing of the measurement session is illustrated in Fig. 1 and their progress is described point by point below.

#### Baseline measures

On the day of inclusion, a questionnaire will be given to each participant to obtain demographic information, note any previous related injury, establish a physical activity profile and question the presence of balance disorders. If any related pain is reported, it will be evaluated on a numerical scale ranging from 0 (no pain) to 10 (worst pain).

Participant weight and height as well as blood pressure will be collected on D0, 7 and 21. Patients will be questioned on any adverse events and pain will be evaluated if necessary.

#### Questionnaire

An SF-36 questionnaire [56] will be distributed before first intervention for self-completion to assess health-related quality of life, allow to characterize the population and possibly to better understand atypical responses to treatment.

#### HRV

Participants will be equipped with a heart rate monitor (Polar RS800) and a heart belt to collect HRV. The watch will be placed on the subjects' wrists and the belt on the chest (under the pectoral muscles for men and under the bra for women). The skin under the sensor will be slightly moistened to ensure good electrical contact and to avoid artifacts. The belt must be tight enough to ensure that the sensor is in perfect contact with the skin but must remain comfortable. Regarding the Coronary Flow Variation (CFV) measurement protocol, a recent study demonstrated that a new Smartphone algorithm, based on R-R interval stabilization analysis, allows CFV measurement in only 5 min, providing results similar to the conventional methodology in the setting of a falls clinic [57]. Thus, a total recording time of 5 min, in the supine position and with spontaneous breathing, will be performed on each patient. Analysis of the HRV data will be performed using Kubios HRV software.

#### Cervical range of motion

Secondly, the patients will be seated in a neutral position, with their backs against the back of the chair, and will perform the cervical range of motion tests. For this, the experimenter will use a measuring tape and the anatomical points previously identified: ear tragus, chin symphysis, anterior angle of the acromion, sternal incisure. The subject will be asked to perform movements of flexion, extension, right rotation, left rotation, right tilt, and left tilt, returning to the neutral position each time. All movements will be performed 3 times. The measurement will retain the difference between the neutral position and the maximum position of the movement [58].

#### Cervical isometric maximal strength

Next, the cervical strength tests will be performed seated too, using a manual dynamometer (Micro FET2, Hoggan, Salt Lake City, USA). To obtain the maximum isometric force of the neck muscles in the 6 movements, a "make test" will be performed: the experimenter will resist the subject's force without trying to exceed it. The subject will be asked to perform progressively and as hard as possible, from the neutral position, a flexion movement (resistance applied under the chin), an extension movement (resistance applied to the back of the occiput), a right and left rotation movement (resistance applied along the horizontal side of the right and left mandibles), and a right and left tilt movement (resistance applied above the right and left ears).

#### Quadriceps strength

The same type of test will be performed to measure the maximal isometric extension force of the dominant quadriceps by applying the dynamometer to the lower tibia and requesting leg extension from a 90° flexion position [59].

#### WS, SPPB test and plantar support

The subject will then be asked to stand up and perform the different steps of the SPPB test following the instructions of the evaluator [60, 61], leading to an overall score out of 12 points. He will perform the 4 m gait measurement part (which will measure WS) equipped with W-INSHOE plantar sensors (Medicapteur, Balam, France). Data will be analyzed using the software of the same name. The sensors are attached to the skin with hypoallergenic tape.

#### Static posturography test

Finally, the static posturography test will be performed under standard conditions using the WIN-POSTURO platform (Medicapteur, Balma, France) allowing the measurement of displacements of the CoP in a static standing position at a frequency of 40 Hz for 25.6 s [62]. The measurement will be repeated 3 times. The data collected will be processed using WIN-POSTURO software (Medicapteur, Balma, France).

### Statistical analysis and sample size

We calculated 20 subjects per group would allow statistical power of 90% to detect a clinically meaningful increase in WS of 0.2 m/s (± 0.2) with an alpha risk of 0.05 and considering a dropout probability of 10%. SPPB score was not used to calculate the sample size because of the ceiling effect reported for individuals who have high functional abilities, although it remains relevant as primary outcome combined with WS [63].

All data will be expressed as mean ± SD. Normality will be verified by an Agostino-Pearson test. Comparison of data between the two groups will be performed with a one-way ANOVA, followed by a Tukey post-hoc test or a Kruskal–Wallis test, and finally by a Dunn post-hoc test. The gender effect will be considered with a two-way ANOVA, followed by a Bonferroni post-hoc test. The significance level was set at p ≤ 0.05. The effect size of Cohen’s *d* will be calculated for all outcomes and classified with 0.20, 0.50 and 0.80 thresholds to interpret small, medium or large effect. Statistical analyses will be performed using GraphPad Prism 6 (GraphPad Software, La Jolla, CA, USA).

## Discussion

### Potential impact and significance

This study could not go beyond the recruitment phase in 2019, interrupted by the COVID pandemic, but it is currently envisaged that the protocol will be reinstated in 2022. Indeed, manual therapy has already shown its benefits in the management of pain and its place as the therapy of choice in the "toolbox" of the senior citizen (64). We believe that it is interesting to evaluate its impact on balance with a view to including it in the therapies offered in the context of fall prevention. We hope that the analysis of the results will show, among other things, a significant improvement of the SPPB score and of WS after MFR on D21. We also hope that the analysis of the other parameters: cervical mobility and strength, HRV, plantar support and static posturography will be sensitive to manual therapy and that the results will improve the understanding of the links between the cervical spine and balance parameters.

We recognize that the application of a defined protocol of MFR techniques is a limitation of this study, as this does not represent the current management in manual therapy. Nevertheless, from a scientific point of view, the effects of such a protocol applied to the senior cervical spine must be determined before being combined with other interventions. Other limitations could be cited, such as the lack of long-term follow-up.

As "first-line" practitioners, manual therapists such as chiropractors or osteopaths must be trained in the detection and management of balance disorders in seniors. We believe that this study will provide an innovative and well-argued point of view on the evaluation and integration of the cervical spine in manual treatment protocols in the context of the risk of falls in the elderly, which can be disseminated to manual therapy students and other related professionals so that they may have a new therapeutic option to offer.

## Data Availability

Not applicable.

## Abbreviations

C: Cervical
CoP: Center of Pressure
CFV: Coronary Flow Variation
D: Day
DO: Diploma in Osteopathy
HRV: Heart rate variability
MFR: Myofascial release
SPPB: Short Physical Performance Battery test
T: Thoracic
TUGT: Time up and go test
WS: Walking speed
